# Chronic skin ultraviolet irradiation induces transcriptomic changes associated with microglial dysfunction in the hippocampus

**DOI:** 10.1186/s13041-022-00989-6

**Published:** 2022-12-21

**Authors:** Kyeong-No Yoon, Yujin Kim, Yidan Cui, Jungeun Ji, Gunhyuk Park, Jin Ho Chung, Yong-Seok Lee, Joon-Yong An, Dong Hun Lee

**Affiliations:** 1 Department of Biomedical Sciences, Seoul National University Graduate School, Seoul, Republic of Korea; 2 Laboratory of Cutaneous Aging Research, Biomedical Research Institute, Seoul National University Hospital, Seoul, Republic of Korea; 3 Institute of Human-Environmental Interface Biology, Medical Research Center, Seoul National University, Seoul, Republic of Korea; 4 Department of Integrated Biomedical and Life Science, Korea University, Seoul, Republic of Korea; 5 BK21FOUR R&E Center for Learning Health Systems, Korea University, Seoul, Republic of Korea; 6 Department of Dermatology, Seoul National University Hospital, Seoul National University College of Medicine, Seoul, Republic of Korea; 7 Herbal Medicine Resources Research Center, Korea Institute of Oriental Medicine, Seoul, Republic of Korea; 8 Institute On Aging, Seoul National University, Seoul, Republic of Korea; 9 Department of Physiology, Seoul National University College of Medicine, Seoul, 03080 Republic of Korea; 10 Neuroscience Research Institute, Seoul National University College of Medicine, Seoul, Republic of Korea; 11 Wide River Institute of Immunology, Seoul National University, Hongcheon, Republic of Korea; 12 School of Biosystem and Biomedical Science, College of Health Science, Korea University, Seoul, Republic of Korea

**Keywords:** Hippocampus, Microglia dysfunction, Ultraviolet irradiation

## Abstract

Recent evidence indicates that ultraviolet (UV) exposure of the skin can affect brain functions such as learning and memory, addictive behavior, and hippocampal neurogenesis. These changes are closely associated with hippocampal function, which plays a pivotal role in learning and memory formation. However, the molecular mechanisms underlying these UV-induced skin-brain interactions remain unclear. To elucidate the molecular signature associated with UV-induced neurobehavioral changes, we analyzed the hippocampal transcriptome in a well-established mouse skin aging model, which showed thickened skin and impaired hippocampal memory. Transcriptome analysis revealed that significantly downregulated genes in UV-irradiated mice are enriched in neuroimmune-related signaling pathways. Furthermore, cell-type analysis showed that DEGs are also enriched in microglia. Consistently, immunofluorescence imaging showed an increased number of Iba1-positive microglia in the hippocampi of UV-irradiated mice. Collectively, our findings highlight that chronic UV irradiation of the skin causes significant changes in the neuroimmune system in the hippocampus, accompanied by microglial dysfunction and cognitive impairment.

**Supplementary Information:**

The online version contains supplementary material available at 10.1186/s13041-022-00989-6.

## Main text

Bidirectional communication between the skin and brain regulates local and global homeostasis in the body [1]. The skin is the first barrier located at the interface with the external environment, and it senses, collects, and responds to environmental stimuli (such as oxidative stress, ultraviolet (UV) and infrared radiation, and pollution) [2]. Skin converts these external stresses into cellular signals. These molecular signals are then transmitted to the brain or other organs and affect the global homeostasis of individuals [1–7]. UV rays are an essential component of sunlight and are considered the most important source of external stress causing extrinsic aging, which is also referred to as photo-aging [3]. Several studies suggest that UV exposure of the skin not only induces skin-related biochemical responses, but also affects signal transduction pathways in other organs such as the brain. For instance, UV exposure increases both skin and brain levels of β-endorphin in mice, which leads to opioid-related antinociception and the subsequent addiction of mice to UV light [8]. Moderate UV exposure increases the blood level of urocanic acid and enhances the learning and memory of the mouse brain through the glutamate biosynthetic pathway [9]. As the primary brain area involved in cognitive function [10], the hippocampus plays a major role in learning and memory formation [11]. Thus, the hippocampus is widely considered an essential target for the investigation of cognitive function. Previously, we reported that repeated UV exposure of the skin decreases hippocampal neurogenesis and synaptic plasticity [1]. Although mounting evidence suggests that UV exposure may induce neurobehavioral changes in the brain, the molecular mediators are largely unknown. Moreover, the cellular players involved in UV-induced neurobehavioral changes have not been explored. To specify the molecular and cellular signatures associated with UV-induced functional changes in the brain, especially in learning and memory, we analyzed the hippocampal transcriptome in chronic UV-irradiated photoaged mice.

**Fig. 1:**
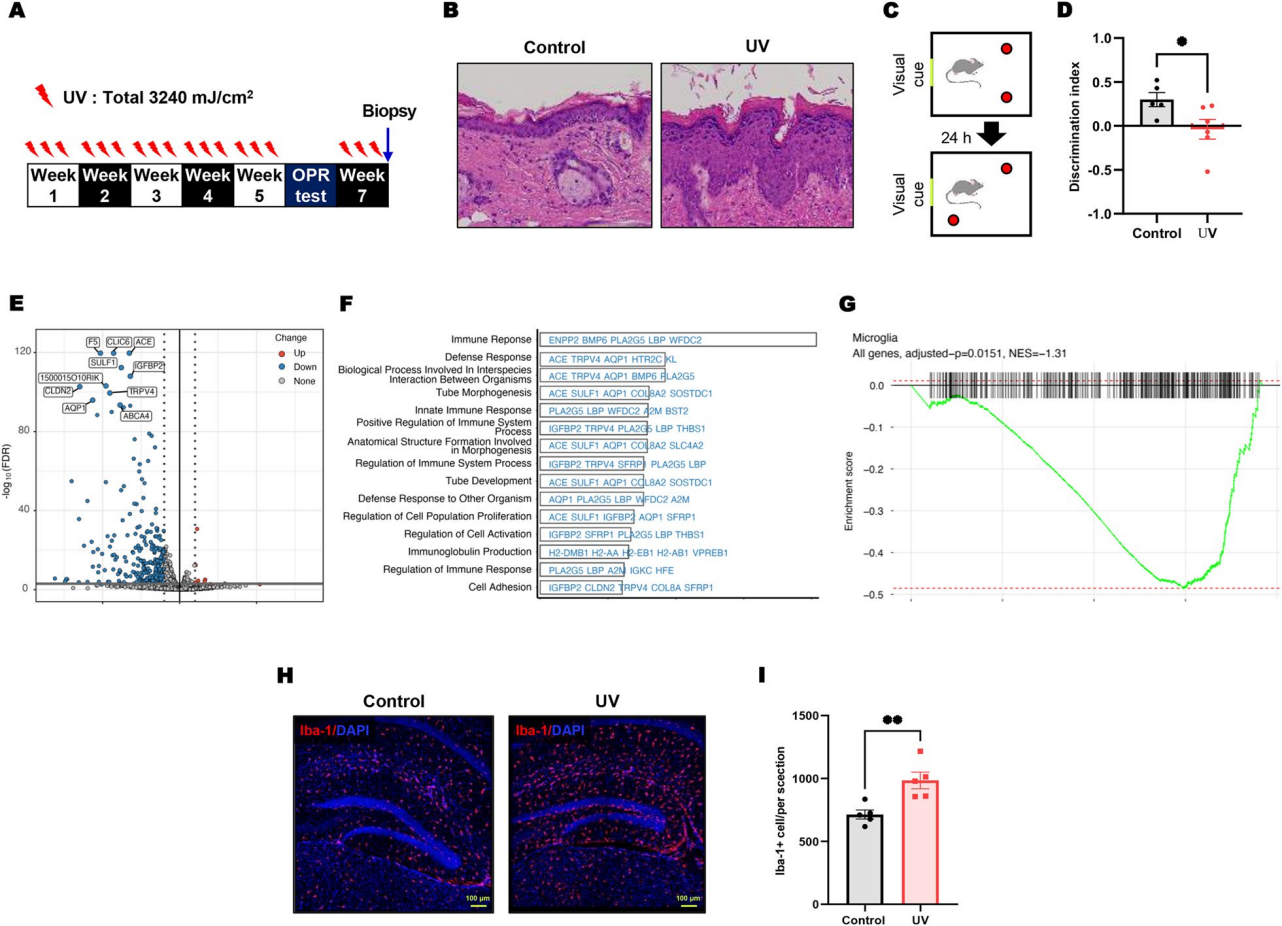
Chronic skin UV irradiation induced hippocampal memory deficit and transcriptomic changes associated with microglial function. **A**–**D** Experimental schedule of UV irradiation given 3 days a week for 6 weeks. After 5 weeks of UV irradiation, the object place recognition test was performed. **B** H&E staining. After 5 weeks of UV irradiation, the discrimination index was calculated as the difference between **C**, **D** the exploring time to novel place object (N) and familiar object (F), divided by the total time exploring both objects (discrimination index = (N − F)/(N + F).*P < 0.05 vs. control group. Each bar represents the mean ± SEM of each group. **E**–**G** Result of differentially expressed gene (DEG) analysis. **E** Volcano plot of UV-DEGs. Y axis refers to the adjusted p-values in − log_10_ scale. X axis refers to the mean expression ratio of UV-irradiated and control mice samples in log_2_ scale. The colors of the dots indicate the direction of regulation (red: up-regulation, blue: downregulation, gray: non-significant regulation). Adjusted p-values lower than 1.0 × 10^–120^ are displayed as 2.0 × 10^–120^ in the figure. Top 10 most downregulated DEGs are marked by larger points and labeled. **F** Functional annotations for UV-DEGs showing the top 15 significantly enriched gene ontology (GO) biological pathways. Top five UV-DEGs ranked by FDR overlapping with leading edge genes are shown, with letter colors indicating the direction of regulation (red: up-regulation, blue: downregulation). **G** Enrichment plot of microglia markers. X axis refers to the rank of genes in descending order of log_2_ fold change values. The vertical black lines on the X axis are the genes overlapping with the markers. The green line shows the cumulative enrichment score, calculated by walking down the gene list, where the score increases if a gene overlaps with the markers and decreases if a gene does not. The red dashed lines indicate the maximal and minimal scores. **H** Representative photographs of Iba-1+ cells in the hippocampal region. **I** The total number of Iba-1+ cells in the hippocampus were quantified in the graph. Each bar represents the mean ± SEM of each group. The asterisks denote a significant difference (**P < 0.01 vs. control group)

We established a skin aging model by sequentially increasing the UV dose three times a week for 6 weeks (Fig. 1A). The dorsal skin of anesthetized mice was UV irradiated in the treated group, and the mice in the control group were anesthetized and sham-irradiated. After UV irradiation for 6 weeks, hematoxylin and eosin (H&E) staining confirmed that the epidermal layers were thickened, indicating that chronic UV irradiation induces skin aging (Fig. 1B). To investigate the effect of UV irradiation on cognitive function, mice were subjected to the object place recognition (OPR) behavior test, which depends on intact hippocampal function [12]. The discrimination index was significantly lower in UV-irradiated mice than in sham-irradiated mice (Fig. 1C, D, *P* = 0.049), indicating that chronic UV irradiation impaired cognitive function (Additional file 1).

To explore the molecular and cellular mechanisms underlying the memory deficit induced by chronic UV exposure to the skin, we performed RNA-seq analysis of the hippocampi from UV- and sham-irradiated mice and identified a total of 338 differentially expressed genes (DEGs), including 10 upregulated and 328 downregulated genes in UV-irradiated mice, compared to sham-irradiated controls (Fig. 1E and Additional file 2: Table S1). To elucidate biological functions perturbed by UV irradiation, we performed pre-ranked gene set enrichment analysis (GSEA) using mouse gene ontology (GO) biological pathway terms provided by the MsigDB collection [13]. Immune-related pathways were significantly enriched for downregulated DEGs, including immune response (GO: 0006955, normalized enrichment score (NES) = − 1.46, false discovery rate (FDR) = 2.2 × 10^–10^), regulation of immune system process (GO: 0002682, NES = − 1.33, FDR = 1.4 × 10^–4^), and immunoglobulin production (GO: 0002377, NES = − 1.71, FDR = 5.6 × 10^–4^) (Fig. 1F and Additional file 2: Table S1). Several genes, including *Pla2g5*, *Lbp*, and *Wfdc2*, that commonly appear in these pathways were significantly downregulated and validated in the UV-exposed mice (Additional file 3: Fig. S1). This suggests that UV exposure reduces the functionality of the neuroimmune system and putatively leads to glia-related disruption in the central nervous system. Thus, we dissected the cell-type enrichment of DEGs using a single-cell transcriptome dataset of the mouse cortex and hippocampus [14]. We collected cell-type-specific marker genes for eight major cell types: hippocampal CA1 pyramidal neurons, interneurons, microglia, astrocytes, oligodendrocytes, endothelial, ependymal, and mural cells. Our GSEA results showed that microglia (NES = − 1.31, FDR = 1.5 × 10^–2^) were significantly enriched for downregulated DEGs in UV-exposed mice (Fig. 1G and Additional file 2: Table S1). Taken together, this suggests that UV exposure leads to a reduction in microglia-specific gene expression and may impair microglial functionality in the neuronal system.

Microglial activation can be determined by various parameters such as cell number, morphology, gene expression, or cytokines. In the hippocampus, it was demonstrated that the extent of microglial cell activation increased with an increase in the “number of microglial cells” [15]. To further investigate microglia-mediated neuroinflammation after chronic UV exposure, we conducted immunohistochemical evaluation of microglial activation. The number of ionized calcium-binding adaptor protein-1(Iba-1)-positive microglial cells in each group were quantified. As indicated in Fig. 1H, I, the number of Iba-1-positive cells was significantly increased in the hippocampal regions of UV-irradiated mice. These results indicate that UV exposure may induce the activation of microglial cells and consequently microglia-mediated neuroinflammation in the hippocampus. It is of note that the expression of Iba-1 was not altered at 6 weeks (Additional file 2: Table S1). The discrepancy between immunostaining and mRNA assays has also been reported in previous studies. Furthermore, immunostaining that can detect changes in the number and morphology of micrmicroglia has been suggested as a sensitive marker for activated microglia [15]. Also, the correlation between downregulation of specific microglia-related genes and microglial activation requires further investigation.

In conclusion, our findings highlight the unambiguous dysfunction of cognitive and neuroimmune systems induced by chronic UV irradiation. By analyzing the hippocampal transcriptome, we first demonstrated that repeated UV exposure of the skin could reduce the expression of neuroimmune system- and microglia-related genes. Moreover, after UV irradiation, microglial cells are activated, which may cause microglia-mediated neuroinflammation in the hippocampus.

## Supplementary Information

**Additional file 1.** Additional Materials and Methods.

**Additional file 2: Table S1.** Raw data of RNA-sequencing analysis.

**Additional file 3: Figure S1.** RT-PCR validation of RNA-seq results. The expression levels of *Pla2g5*, *Lbp*, and *Wfdc2* were analyzed in the hippocampus via quantitative reverse transcription-polymerase chain reaction testing. The bars represent the mean ± SEM of each group. The asterisks denote a significant difference (*P < 0.05, **P < 0.01 vs. Control group. Control, n = 4 mice, UV, n = 5 mice).

## Data Availability

All data generated or analyzed during this study are included in this article and its Additional files.

## Abbreviations

UV: Ultraviolet
OPR: Object Place Recognition test
DEGs: Differentially Expressed Genes
GO: Gene ontology
GSEA: Gene set enrichment analysis
Iba-1: Ionized calcium-binding adapter molecule-1

## References

[1] Han M, Ban J-J, Bae J-S, Shin C-Y, Lee DH, Chung JH (2017). UV irradiation to mouse skin decreases hippocampal neurogenesis and synaptic protein expression via HPA axis activation. Sci Rep.

[2] Marek-Jozefowicz L, Czajkowski R, Borkowska A, Nedoszytko B, Żmijewski MA, Cubała WJ, Slominski AT (2022). The brain–skin axis in psoriasis—psychological, psychiatric, hormonal, and dermatological aspects. Int J Mol Sci.

[3] Debacq-Chainiaux F, Leduc C, Verbeke A, Toussaint O (2012). UV, stress and aging. Dermatoendocrinology.

[4] Hart PH, Gorman S, Finlay-Jones JJ (2011). Modulation of the immune system by UV radiation: more than just the effects of vitamin D?. Nat Rev Immunol.

[5] Roosterman D, Goerge T, Schneider SW, Bunnett NW, Steinhoff M (2006). Neuronal control of skin function: the skin as a neuroimmunoendocrine organ. Physiol Rev.

[6] Slominski A, Wortsman J (2000). Neuroendocrinology of the skin. Endocr Rev.

[7] Slominski AT, Zmijewski MA, Plonka PM, Szaflarski JP, Paus R (2018). How UV light touches the brain and endocrine system through skin, and why. Endocrinology.

[8] Fell GL, Robinson KC, Mao J, Woolf CJ, Fisher DE (2014). Skin β-endorphin mediates addiction to UV light. Cell.

[9] Zhu H, Wang N, Yao L, Chen Q, Zhang R, Qian J, Hou Y, Guo W, Fan S, Liu S (2018). Moderate UV exposure enhances learning and memory by promoting a novel glutamate biosynthetic pathway in the brain. Cell.

[10] Maurer AP, Nadel L (2021). The continuity of context: a role for the hippocampus. Trends Cogn Sci.

[11] Zhang J-X, Chen D-B, Dong Q, Zhao Z-D (2016). Identifying a set of influential spreaders in complex networks. Sci Rep.

[12] Lee Y-S, Silva AJ (2009). The molecular and cellular biology of enhanced cognition. Nat Rev Neurosci.

[13] Liberzon A, Birger C, Thorvaldsdóttir H, Ghandi M, Mesirov JP, Tamayo P (2015). The molecular signatures database hallmark gene set collection. Cell Syst.

[14] Zeisel A, Muñoz-Manchado AB, Codeluppi S, Lönnerberg P, La Manno G, Juréus A, Marques S, Munguba H, He L, Betsholtz C (2015). Cell types in the mouse cortex and hippocampus revealed by single-cell RNA-seq. Science.

[15] Wittekindt M, Kaddatz H, Joost S, Staffeld A, Bitar Y, Kipp M, Frintrop L (2022). Different methods for evaluating microglial activation using anti-ionized calcium-binding adaptor protein-1 immunohistochemistry in the cuprizone model. Cells.

