# Supplementary material for: Chronic skin ultraviolet irradiation induces transcriptomic changes associated with microglial dysfunction in the hippocampus
Source: Mol Brain. 2022 Dec 21;15:102. doi: 10.1186/s13041-022-00989-6 (PMC9768969; doi:10.1186/s13041-022-00989-6)
Supplement: Supplementary file 1 — Additional file 1. Additional Materials and Methods. [file 13041_2022_989_MOESM1_ESM.docx]

**Additional File 1**

**Chronic skin ultraviolet irradiation induces transcriptomic changes associated with microglial dysfunction in the hippocampus**

Kyeong-No Yoon^1,2,3,†^, Yujin Kim^4,5,†^, Yidan Cui^2,3,6^, Jungeun Ji^4,5^, Gunhyuk Park^7^, Jin Ho Chung^1,2,3,6,8^, Yong-Seok Lee^9,10,11,*^, Joon-Yong An^4,5,12,*^, Dong Hun Lee^2,3,6*^

^†^ These authors equally contributed to this work.

^*^ Corresponding authors: Yong-Seok Lee (yongseok7@snu.ac.kr), Joon-Yong An ([joonan30@korea.ac.kr](mailto:joonan30@korea.ac.kr)), and Dong Hun Lee ([ivymed27@snu.ac.kr](mailto:ivymed27@snu.ac.kr)).

**This file includes:**

**Additional Materials and Methods**

**Additional Materials and Methods**

**Animal experiment**

Six-week-old female SKH-1 hairless mice were purchased from Orient Bio (Seongnam, Korea). The animals were allowed ad libitum access to food and were adapted for a week before the study. All experimental protocols were approved by the Institutional Animal Care and Use Committee (No. 20–0271) of the Biomedical Research Institute at Seoul National University Hospital and were performed in accordance with the relevant guidelines and regulations. SKH-1 hairless mice were randomly allocated to two groups.

**UV irradiation**

UV irradiation was performed using TL20W/12RS UV lamps (Philips, Eindhoven, Netherlands) with an emission spectrum between 275 and 320 nm. UVC (< 290 nm) wavelengths were blocked using a Kodacel filter (TA401/407; Kodak, Rochester, NY, USA) which was placed 2 cm in front of the UV lamp. The UV intensity was measured using a UV meter (Model 585100, Waldmann, Villingen-Schwenningen, Germany).

**Object Place Recognition test (OPR test)**

The OPR test was performed using a modified method after UV irradiation for 5 weeks. Briefly, the test setup consisted of an opaque plastic apparatus, and the procedure consisted of four phases: handling, habituation, training, and testing. Before the behavior test, a mouse was accustomed to handling for 5 min a day for 4 days to reduce anxiety (handling phase). The mouse was allowed to freely navigate the chamber without objects for 15 min to acclimatize it to the environment (habituation phase). Twenty-four hours later, the mouse was exposed to the two objects for 10 min (training phase). After training, one object was moved to a new location to evaluate long-term memory, and the mouse was placed in the chamber and observed for 5 min (test phase). The time at which the mouse showed interest in the object at a new location was measured. The testing sessions were recorded using a video camera and analyzed by a blinded examiner. Object recognition was defined as the case in which the mouse’s nose touched the object or entered within 2 cm of it. The discrimination index was calculated as the difference between the time spent exploring the novel place object and the familiar object divided by the total time spent exploring both objects.

**RNA sequencing analysis**

Bulk RNA samples were collected from the mice hippocampi. We obtained two UV-exposed and two control mice samples. Differential gene expression analysis was performed using the DESeq2 R package [1]. Genes with log_2_ fold change > 1 and adjusted p-value < 0.05 were selected as significantly regulated under UV exposure.

**Gene ontology (GO) analysis**

To evaluate the functional consequences of UV exposure, GO analysis was conducted via pre-ranked gene set enrichment analysis (GSEA) of all expressed genes onto GO biological pathway terms using fgsea R package [1]. The GO database was obtained from MsigDB mouse GO gene sets and a subset of biological pathways (7,775 pathways) was used [2][3]. Genes were pre-ranked by log_2_ fold change values, and pathway terms with a false discovery rate (FDR) < 0.05 were considered significant.

**Gene set enrichment analysis (GSEA)**

To examine the cell-type-specific signatures in UV-exposed mice samples, we applied GSEA to all genes from RNA sequencing using the fgsea R package. Cell type-specific markers were obtained from a single-cell dataset of the mouse cortex and hippocampus [4]. Among the nine cell types, the S1 pyramidal cell type was removed because no hippocampal cells were present in the cell type in the reference study. Markers that were not expressed in the sample were excluded. Genes were pre-ranked by log_2_ fold change values, and cell types with FDR < 0.05 were considered significant.

**Iba-1 Immunohistochemistry**

Immunohistochemistry for Iba-1 was performed using the free-floating technique. One series was randomly selected and stained with antibodies against Iba-1 (019-19741, Wako, Osaka, Japan). The free-floating slices were incubated for two days at 4 °C with the primary antibodies in a diluent buffer comprising 1% bovine serum albumin (BSA) (9048-46-8, Sigma-Aldrich, St. Louis, MO, USA) and 1% Triton X-100 in 0.1 M phosphate buffer. After five washes with PBS, the sections were incubated for 3h with the following secondary antibodies in dilution buffer: Alexa Fluor 594 goat anti-rabbit IgG (A11012, Invitrogen). After three washes with PBS, the sections were incubated with DAPI (2 μg/ml) for 5 min and mounted in X-CLARITY Mounting Solution (C13101, VitaScientific, VA, USA). Immunofluorescence images of sections were captured using a confocal laser-scanning microscope (A1Rsi, Nikon, Tokyo, Japan).

**References**

1. Korotkevich G, Sukhov V, Budin N, Shpak B, Artyomov MN, Sergushichev A: **Fast gene set enrichment analysis.** *BioRxiv* 2021**:**060012.

2. Consortium GO: **The gene ontology resource: 20 years and still GOing strong.** *Nucleic acids research* 2019, **47:**D330-D338.

3. Liberzon A, Birger C, Thorvaldsdóttir H, Ghandi M, Mesirov JP, Tamayo P: **The molecular signatures database hallmark gene set collection.** *Cell systems* 2015, **1:**417-425.

4. Zeisel A, Muñoz-Manchado AB, Codeluppi S, Lönnerberg P, La Manno G, Juréus A, Marques S, Munguba H, He L, Betsholtz C: **Cell types in the mouse cortex and hippocampus revealed by single-cell RNA-seq.** *Science* 2015, **347:**1138-1142.
