# Supplementary figures and images for: Chronic skin ultraviolet irradiation induces transcriptomic changes associated with microglial dysfunction in the hippocampus
Source: Mol Brain. 2022 Dec 21;15:102. doi: 10.1186/s13041-022-00989-6 (PMC9768969; doi:10.1186/s13041-022-00989-6)

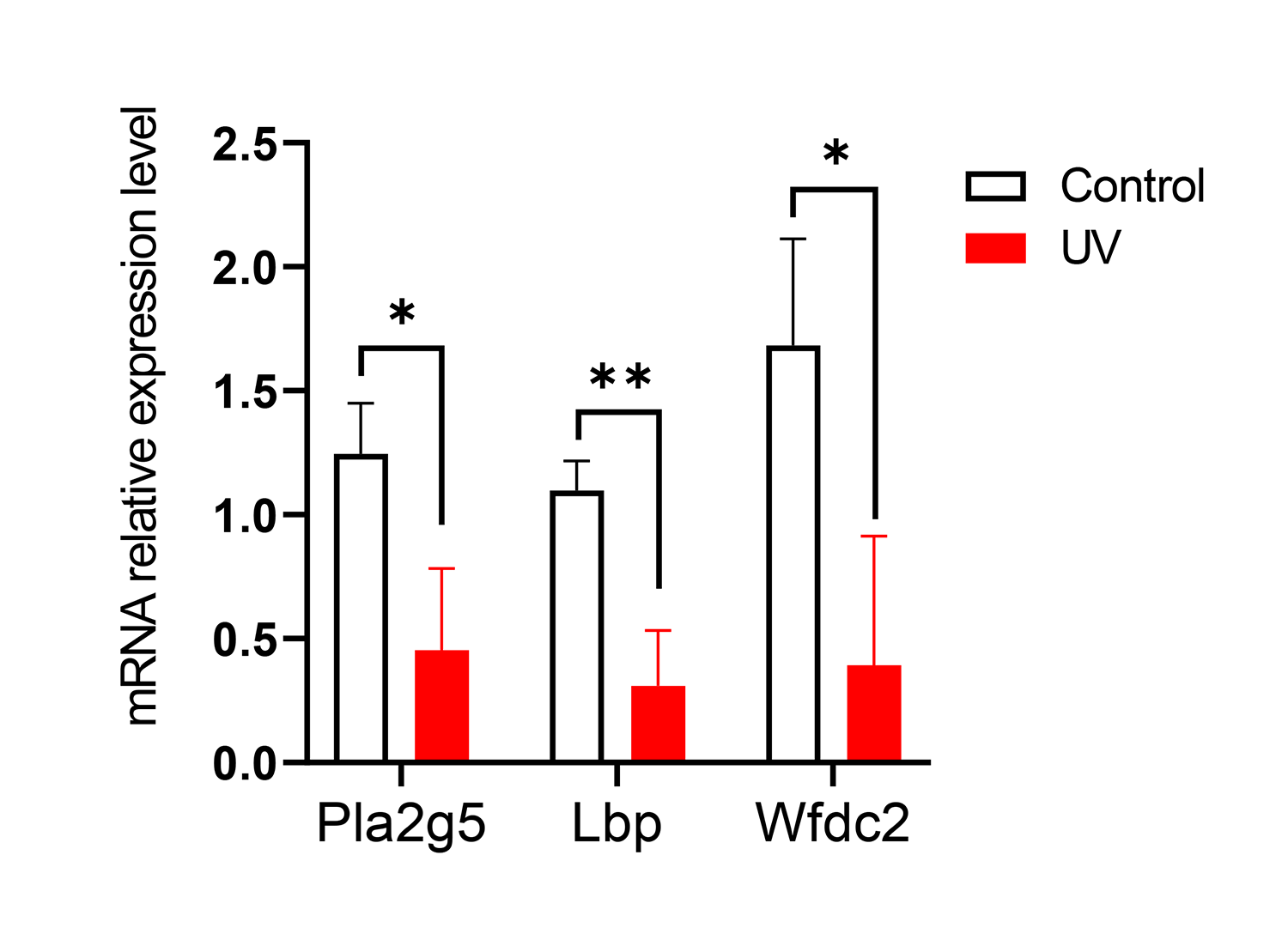

Supplement: Supplementary file 3 — Additional file 3: Figure S1. RT-PCR validation of RNA-seq results. The expression levels of Pla2g5, Lbp, and Wfdc2 were analyzed in the hippocampus via quantitative reverse transcription-polymerase chain reaction testing. The bars represent the mean ± SEM of each group. The asterisks denote a significant difference (*P < 0.05, **P < 0.01 vs. Control group. Control, n = 4 mice, UV, n = 5 mice). [file 13041_2022_989_MOESM3_ESM.tif]
